# The correctness and completeness of documentation of parameters on the partographs used by midwives in primary healthcare facilities in midwestern Uganda: A retrospective descriptive study

**DOI:** 10.1002/nop2.1383

**Published:** 2022-09-27

**Authors:** Archbald Bahizi, Clement Munguiko, Enos Mirembe Masereka

**Affiliations:** ^1^ Department of Nursing and Midwifery, School of Health Sciences Mountains of the Moon University Fort Portal Uganda; ^2^ Uganda Blood Transfusion Services Ministry of Health Kampala Uganda; ^3^ Department of Nursing, School of Health Sciences Soroti University Soroti Uganda; ^4^ Department of Nursing and Midwifery, School of Medicine Kabale University Kabale Uganda

**Keywords:** care, completeness, correctness, intrapartum, partograph

## Abstract

**Aim:**

This study was conducted to determine the correctness and completeness of documentation of partographs.

**Design:**

This was a retrospective descriptive study.

**Methods:**

We included 365 partographs of deliveries conducted from January 1st to October 31st 2019. Data were collected using a checklist and analysed descriptively. The study based on 13 partograph parameters.

**Results:**

About 8–13 parameters were correctly documented in 71.5 % of the partographs. About 38.9%, 24.7%, 99.7%, 22.5% and 16 % of the partographs had no documentation of obstetric risk factors, foetal heart rate, colour of liquor, uterine contractions and cervical dilatation respectively. About 12.1% of the cervicographs crossed the action line and 61.4% of partographs where cervicographs crossed the action line had no documentation of action(s) taken.

## 1 INTRODUCTION

Maternal mortality remains a burden in developing countries. Almost all (94%) of maternal deaths occur in developing countries, and 65% occur in Africa (World Health Organization, 2019a). The partograph is highly effective in reducing complications from obstructed and prolonged labour such as uterine rupture, postpartum haemorrhage and sepsis. Prolonged and obstructed labour contribute about 8–10 percent of maternal deaths (Nour, 2008; World Health Organization, 2019b). These deaths can be averted if partographs are consistently and correctly used during monitoring of mothers experiencing labour.

## 2 BACKGROUND

The partograph is a labour monitoring tool recommended by the World Health Organization (WHO) for use in monitoring the progress of labour, the wellbeing of the mother and the baby during the active stage of labour. The partograph informs when timely intervention is required (Bedwell et al., 2017) and improves the labour outcomes of both the mother and the baby. The execution of an appropriate intervention following abnormal findings on a partograph could reduce the rates of obstructed labour, prolonged labour, obstetric fistula, foetal birth trauma, stillbirth, foetal hypoxia and neonatal death (Gans‐Lartey et al., 2013).

Despite the ability to improve labour outcomes and prevent maternal and neonatal mortality, the partograph has not been widely and appropriately used especially in low and middle income countries (Chaturvedi et al., 2015; Ollerhead & Osrin, 2014). Findings from studies in Ethiopia showed that, 31.0–42.7 percent of the obstetric care givers at public health institutions did not use the partograph to monitor mothers during labour and some of those who used, were uncertain on whether their partographs implicitly reflected the condition of the mothers they monitored (Hagos et al., 2020; Yisma et al., 2013).

The qualities of an appropriately used partograph include accuracy, correctness and completeness in documentation of parameters on the partograph. However, in many settings, most filled partographs have not met these standards and studies have shown that partographs are inaccurately, incompletely and incorrectly filled and may not implicitly reflect the condition of the mother being monitored. For instance; a study in Bangladesh showed that, foetal heart rate and cervical dilatation were incorrectly recorded in 39 percent and 30 percent of the partographs, respectively (Khan et al., 2018). Another study in Ghana showed that; only 25.6 percent of partographs were adequately completed with the highest compliance rate noted with documentation of cervical dilatation and lowest compliance rate was of documentation of foetal heart rate (Gans‐Lartey et al., 2013). In Malawi, study findings revealed that more than half of the partographs had no recording of moulding, foetal heart rate and descent (Mandiwa & Zamawe, 2017) and one of the studies in Uganda indicated that 61 percent of the partographs had incomplete recording of monitoring of foetal condition, maternal condition and labour progress (Mukisa et al., 2019).

Documentation of partographs has always been integral to midwifery practice in many settings including Uganda (Mathibe‐Neke et al., 2013). The partograph is used in expectant women anticipated to have normal vaginal delivery. These women are majorly monitored by midwives and thus, making midwives the major users of partographs among all other skilled birth attendants. Additionally, when there is a deviation in the course of labour, the midwife will institute an appropriate intervention. Some interventions will require collaborative action with an obstetrician. However, inappropriate use of partograph that includes inaccuracy, incorrectness and incompleteness of documentation of parameters, is common and has been attributed to overwhelming numbers of expectant mothers, and congestion in the maternity wards, other responsibilities assigned to midwives, presence of other monitoring tools to be filled, limited skills among midwives, inadequate equipment and supplies and the state of the mother at presentation to labour suit (Mukisa et al., 2019). The correctness and completeness of documentation of parameters on the partograph impacts on its interpretation, timely decision‐making and emergency intervention prioritization (Mandiwa & Zamawe, 2017). It is, therefore, important that partographs are accurately, correctly and completely filled.

### 2.1 Study aim

This study was conducted to determine the correctness and completeness of documentation of partographs and whether action is undertaken when the cervicograph moves towards the action line in high client volume health facility settings in Midwestern Uganda.

## 3 THE STUDY

### 3.1 Study setting and population

This study was conducted from six (Clement et al., 2019) selected high client volume health facilities in Kamwenge district, Western Uganda. Health facilities that were selected offered outpatient and inpatient maternal services including normal deliveries, management of complicated labour, family planning and newborn care. Kamwenge is one of the districts in Uganda that has been earmarked by government as one of the well performing districts in terms of midwifery and newborn services. We retrieved partographs of pregnant women who had delivered from 1 January–31 October 2019 and reviewed them in November 2019.

### 3.2 Study design

The study used a retrospective descriptive survey design to understand correctness and completeness of recording of parameters on the partographs and its statistical significance in signalling prompt interventions among pregnant women whose cervicographs moved towards or crossed the action line in active labour.

### 3.3 Sample size and sampling technique

The sample size was determined using the Kish Leslie formula. *P,* the proportion of incomplete partographs in Ugandan hospitals, was set at 61% (Mukisa et al., 2019), *Z* (the value from standard normal distribution) at 95% Confidence Interval (CI) was set at 1.96. *e* (the margin of error) was set at 5% (0.05) to arrive at *N* (the actual sample size) of 365 partographs. Health centres with more than 30 deliveries a month were purposively selected, basing on this, a total of six (Clement et al., 2019) health facilities were sampled. Partographs for mothers who delivered from 1 January–31 October 2019 were collected from the different study health centres and were randomly arranged. A total of 730 partographs were collected. The first partograph that was included in the study was randomly selected and the rest of the partographs were selected from the pull using a sampling interval of two. This was done until a total of 365 partographs were sampled. Samples of partographs obtained per health centre were as follows; Rukunyu (80), Ntara (40), Mahyoro (35), Rwamwanja (148), Padre pio (32) and Kamwenge (30).

### 3.4 Data collection process

Data on correctness and completeness of recording of parameters on partographs and its statistical significance in signalling prompt interventions among pregnant women in active labour were collected and recorded on predetermined checklist. We adapted and modified the partograph audit tool developed by the Uganda Ministry of Health (MoH) to develop the study checklist. The checklist had three sections. The first section collected data on socio‐demographic and obstetrical variables of pregnant mothers such as the health facility name, inpatient number, age, gravida, para, date and time of admission, risk factors and weeks of gestation. The second section collected data on foetal heart monitoring, state of membranes and progress of labour (cervical dilatation, foetal head descent and uterine contractions). The third section recorded data on actions that were taken and foetal‐maternal outcomes. In this study, correctness and completeness of recordings of parameters on partographs was determined by appropriate documentation of 13 (Mandiwa & Zamawe, 2017) variables. These were; assessment of the mother for potential risk factors, recording of foetal heart rates every half hour, recording of the intactness or rupture of membranes, recording of colour of liquor upon rupture of membranes, recording of foetal head moulding, recording of cervical dilation every four (Bolbol‐Haghighi et al., 2015) hourly, recording of uterine contractions every half hourly, recording of foetal descent every two hourly, recording of maternal temperature every 4‐hourly, recording of maternal pulse rate every half hourly, recording of maternal blood pressure every 4‐hourly, urine testing for proteins and a recording of actions taken when needed. All the 13 variables were scored with either Yes (1) or No (0). The resultant score was, therefore, a range from 0–13. In this study, we, therefore, considered appropriate (accuracy, correctness and completeness) use of partograph for scores from 8–13.

### 3.5 Data analysis

Data was analysed using SPSS version 20. Maternal demographic characteristics, correctness and completeness of partograph and its statistical significance in signalling prompt action among pregnant women in active labour were analysed using descriptive statistics.

### 3.6 Ethical considerations and protection of study participants

In order to ensure quality of research findings, we sought ethical clearance from a local Research and Ethics Committee (REC) at Mountains of the Moon University. Further consent was sought from Kamwege District Administration and from in‐charges of selected health facilities and maternity wards. We pretested the data collection tool at one of the health facilities with similar settings in Kabarole district. Any areas in the checklist that needed improvement were updated before commencement of the study.

## 4 RESULTS

### 4.1 Socio‐demographic characteristics

In this study, a total of 365 filled partographs were studied. Majority of women (89.0%) who were monitored using partograph were aged 18–34 years and were not married (55.6%) [Table 1].

**TABLE 1:** Socio‐demographic characteristics of women whose partographs were studied in midwestern Uganda

Variable	Frequency *N* = 365	Percentage
Age		
<18 years	14	3.9
18–34 years	325	89.0
≥35 years	26	7.1
Gravidity		
≤4 pregnancies	277	75.9
>4 pregnancies	88	24.1
Marital status		
Single	203	55.6
Married	162	44.4
Religion		
Muslim	80	21.9
Christian	285	78.1
Education level		
None	115	31.5
Educated	250	68.5
Occupation		
Informal employment	280	76.7
Formal employment	85	23.3

### 4.2 Correctness and completeness of documentation of parameters on the partograph and documentation of action taken among pregnant women in active labour in Kamwenge district midwestern Uganda

In this study, about a third of the partographs (38.9%) did not have recording of the obstetric risk factors and maternal blood pressure (34.0%). About a quarter of partographs (24.7%) and nearly all (99.7%) did not have recording of the foetal heart rate and colour of liquor, respectively. Almost a quarter (22.5%) and in 16.4% of the partographs, there was no recording of uterine contractions and cervical dilatation, respectively. About 12.1 percent of the cervicographs moved towards or crossed the action line. About health worker's prompt action onto partograph signals, the study revealed that almost two thirds (61.4%) of pregnant women in active labour whose cervicographs moved towards or crossed action line had no documentation of corresponding action that was taken [Table 2].

**TABLE 2:** Correctness and completeness of documentation of parameters on the partograph and documentation of action taken among pregnant women in active labour

	Variable	Frequency (*N* = 365)	Percentage
1	Obstetric risk factors		
	Yes	223	61.1
	No	142	38.9
2	Foetal heart rate		
	Yes	275	75.3
	No	90	24.7
3	State of membranes		
	Yes	308	84.4
	No	57	15.6
4	Colour of liquor		
	Yes	1	0.3
	No	364	99.7
5	Moulding		
	Yes	301	82.5
	No	64	17.5
6	Cervical dilatation		
	Yes	305	83.6
	No	60	16.4
7	Foetal descend		
	Yes	296	81.1
	No	69	18.9
8	Contractions		
	Yes	283	77.5
	No	82	22.5
9	Vitals–Temperature		
	Yes	269	73.7
	No	96	26.3
10	Vitals–Pulse		
	Yes	251	68.8
	No	114	31.2
11	Vitals–BP		
	Yes	241	66.0
	No	124	34.0
12	Urine tested		
	Yes	181	49.6
	No	184	50.4
13	Cervicograph moved towards or crossed the alert line		
	Yes	80	21.9
	No	285	78.1
	Cervicograph moved towards or crossed the action line		
	Yes	44	12.1
	No	321	87.9
14	Action taken was documented (*N* = 44)		
	Yes	17	38.6
	No	27	61.4

### 4.3 Overall correctness and completeness of documentation of parameters on the partograph in Kamwenge district in midwestern Uganda

**FIGURE 1:**
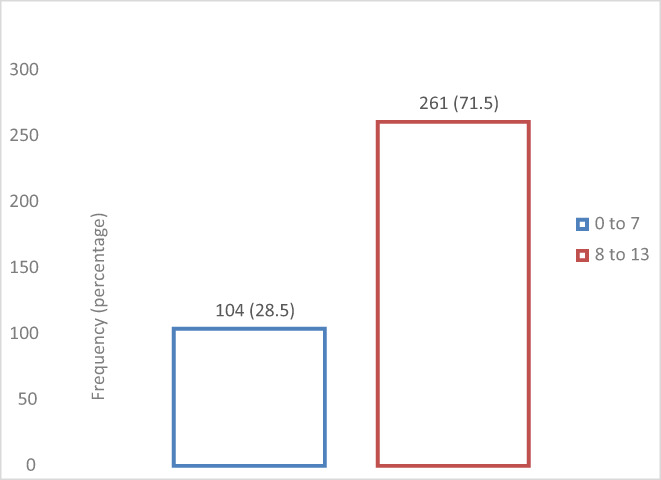
Overall correctness and completeness of documentation of parameters on the partograph in midwestern Uganda

After organizing partograph recording in two categories, we found that 71.5% of the partographs had 8–13 parameters correctly and completely recorded [Figure 1].

## 5 DISCUSSION OF RESULTS

From this study, we found that about half (55.6%) of the pregnant women in labour were not married. High proportion of unmarried pregnant women found in this study is similar to the findings by Clement et al. (2019). Single marital status compounded by the young age, poses a statistically significant risk onto maternal‐neonatal wellbeing of pregnant women and consequently labour outcomes. Single marital status could mean that a statistically significant proportion of pregnant women had unintended pregnancies while majority of them lacked robust emotional, psychological and financial support. Lack of emotional and psychological support has been found to be one of the causes of abnormal pregnancy and labour patterns (Harris & Ayers, 2012).

Usage of partograph allows for early detection of abnormal labour such that a prompt intervention is sought (Dalal & Purandare, 2018). This, however, is possible only when pregnant women are correctly monitored, all parameters on the partograph are correctly recorded and interpreted (Dalal & Purandare, 2018). Our study revealed that foetal heart rate was not recorded in 24.7 percent of the partographs and blood pressure was not recorded in 34.0 percent of the partographs. Surprisingly, our study also revealed that cervical dilatation was not recorded in 16.4 percent of partographs, yet parameters such as state of membranes, colour of liquor, moulding and interpretation of the effect of contractions on labour progress entirely depend on the robustness of this variable. Findings from this study are similar to what other studies established. A study in Bangladesh by Khan et al. 2018 also found that foetal heart rate, moulding and cervical dilatation were not recorded in 39.0 percent, 80.0 percent and 30.0 percent of all partographs, respectively. Similar to findings in a Ugandan study found that foetal heart rate was documented in 62 percent, cervical dilatation in 57 percent, pulse in 20 percent and blood pressure in 35 percent of mothers in active labour (Namwaya et al., 2017). In Uganda, Ayebare et al. (2020) in their study found coinciding results. Since the proportion of women who get complications while giving birth is usually small, it is, therefore, imperative that all women are fully and timely monitored in order to detect such complications. From our study, we are convinced that such parameters might have not been documented on the partograph because they were not monitored during labour. This consequently indicates that a statistically significant proportion of pregnant women in labour were more liable to developing maternal and foetal complications without being detected early. This further meant that such women were more likely not to receive timely intervention, which puts their lives and of their babies at grave risk.

From our study, we found that almost two thirds of pregnant women in labour whose cervical dilatation monitoring graphs moved towards or crossed action line had no corresponding immediate action documented. Monitoring of mothers until the cervical graph moves to the right of the action line has been observed in other studies (Bolbol‐Haghighi et al., 2015). This could be because some midwives lack corresponding knowledge and skills and end up continuously monitoring the mother even passed the action line. Others could be due to un‐documented actions that were actually instituted. Lack of immediate action puts the life of both women and their unborn babies in danger and contribute to poor labour outcomes.

## 6 CONCLUSION AND RECOMMENDATIONS

In this study, about 8–13 parameters were correctly and completely recorded in only 71.5% of the partographs. The level of correctness and completeness of recordings of vital parameters on the partograph in Uganda still remains low. Even though partograph still remains a single labour monitoring tool recommended by World Health Organization, majority of health workers do not use findings presented by the tool to make timely lifesaving interventions. We recommend further pre‐service and in‐service training for midwives on assessment of mothers during labour, documentation of parameters on the partograph and appropriate interpretation to guide prioritization of lifesaving interventions during active labour.

## AUTHOR CONTRIBUTIONS

Archbald Bahizi, Clement Munguiko and Enos Mirembe Masereka conceived this study, collected, analysed the data and wrote the manuscript. All authors read and approved the manuscript. All authors have agreed on the final version and meet at least one of the following criteria [recommended by the ICMJE (http://www.icmje.org/recommendations/)]:

- substantial contributions to conception and design, acquisition of data or analysis and interpretation of data.
- drafting the article or revising it critically for important intellectual content.

## CONFLICT OF INTEREST

Authors declare no competing interests in this study.

## Data Availability

All data for this study shall be availed whenever requested by editorial team, reviewers and other users. The data set can be accessed by sending a request to bahiziarchbald@gmail.com
